# A Case Series Study of Weekly or Fortnightly Transcranial Magnetic Stimulation (TMS) in Major Depressive Disorder

**DOI:** 10.3390/brainsci14050415

**Published:** 2024-04-24

**Authors:** Yvonne Turnier-Shea, Gregory M. Peterson, Marzena Rybak, Saxby Pridmore

**Affiliations:** 1 Hobart TMS, Bellerive, TAS 7018, Australia; turniershea@hotmail.com (Y.T.-S.); mrybak@netspace.net.au (M.R.); s.pridmore@utas.edu.au (S.P.); 2 School of Pharmacy and Pharmacology, University of Tasmania, Hobart, TAS 7001, Australia; 3 Discipline of Psychiatry, University of Tasmania, Hobart, TAS 7001, Australia

**Keywords:** transcranial magnetic stimulation, major depressive disorder, maintenance transcranial magnetic stimulation, preservation transcranial magnetic stimulation, outcomes, case series

## Abstract

Background: Major depressive disorder (MDD) is frequently chronic and relapsing. The use of maintenance or continuation transcranial magnetic stimulation (TMS) has received clinical and some research support. Objective: To conduct a case series study to report the outcomes of once-weekly (OW) or once-fortnightly (OF) continuation TMS in a real-life setting. Methods: We offered OW or OF TMS sessions to patients with MDD in remission or partial remission/relapse. Results: Ten patients received OW TMS and four received OF TMS, for 8 to 46 weeks. No patients in either group who were in remission or partial remission at baseline experienced a relapse. Improvements in HAMD6 and CGI-S scores were statistically significant or of borderline significance for the total sample and the OW group. Conclusions: This naturalistic, open-label observational study indicates that OW TMS is effective as maintenance therapy in MDD, while also offering some support for OF TMS maintenance in preventing relapse.

## 1. Introduction

Major depressive disorder (MDD) is a distressing, incapacitating condition which frequently follows a chronic, relapsing course. Accordingly, all forms of acute treatment, including transcranial magnetic stimulation (TMS), have been extended beyond the acute treatment phase, in attempts to sustain improvements [1]. There is considerable evidence to support TMS for patients with MDD who have not responded to or have only partially responded to antidepressants [2]. However, the role of maintenance TMS is less clear [2,3] and specialist TMS groups have used different maintenance protocols. A systematic review coined the term “preservation TMS”, which was defined as “TMS used to sustain a clinical response” [3].

One form of preservation TMS is “cluster maintenance” (CM-TMS) [4]; when a TMS-induced remission is followed by relapse, an approach is to secure another TMS-induced remission and follow this with “clusters” of five TMS treatments delivered over 3 to 5 days—commencing one month following completion of the successful acute course, with further clusters administered at about monthly or longer intervals. We conceptualised CM-TMS as a form of early relapse treatment [5].

A simpler form of preservation TMS consists of continued sessions after a successful acute course, with single treatment sessions provided according to local schedules [6,7,8]. A meta-analysis found that one TMS session each week, fortnight or month may sustain improvement [9]. However, the group which provided one treatment per month did not find the practice to be beneficial [6] and a systematic review questioned the effectiveness of two or fewer sessions per month [10]. Thus, opinions differ as to whether fortnightly treatment is effective in the prevention of relapse.

We had long provided CM-TMS. Some patients found five treatment sessions over 3 to 5 days every month to be onerous and asked for a form of continuation therapy. As some research indicated that once-weekly (OW) and once-fortnightly (OF) TMS are effective in preservation [7,8,9,10], we agreed. We favoured OW TMS, but provided OF TMS to patients who indicated they were unable to afford the more frequent form (the Australian Government Medical Benefits Schedule (MBS) does not include rebates for maintenance TMS [11], so patients are required to self-fund the treatment).

In their systematic review, Wilson et al. [3] noted the relative lack of evidence-based information on preservation TMS protocols, which vary significantly, and the need for more research. Patients at our clinic were advised that the research base of OW and OF TMS is modest, but that it is supported by experts worldwide [3,7,8,9,10]. Our objective in this case series study was to build on the research base and report the outcomes of OW and OF continuation TMS in a real-life setting.

## 2. Materials and Methods

We provided continuation TMS, in the form of OW or OF sessions, to patients experiencing relapsing MDD. All patients were adults who had suffered chronic, relapsing MDD for more than a decade, and had responded (temporarily) to acute TMS. Exclusion criteria included alcohol and drug problems, other severe psychiatric co-morbidity and conditions carrying significant risk of seizure. They presented serially, beginning in January 2023; thus, the lengths of treatment differed. TMS was provided with MagPro R30 devices (MagVenture; Lucernemarken 15, DK-3520 Farum, Denmark). All patients were stimulated at 110% of resting motor threshold, and according to their individual protocol. Some patients were treated at the left dorsolateral prefrontal cortex with 10 Hz, 4 s trains, 75 trains per session; others were treated at the right dorsolateral prefrontal cortex with 1 Hz, 1800 pulses. Patients were allowed to switch from one protocol to the other according to clinical factors and their preference.

Quantitative assessments were made at commencement and at each treatment presentation. The six-item Hamilton depression rating scale (HAMD6) was the principal measure [12,13]. The Clinical Global Impression-Severity (CGI-S) scale was the secondary measure [14,15]. Both tools are valid measures of the severity and response to treatment in MDD [16,17]. HAMD6 scores of <4 indicate remission, >7 indicate full relapse, and scores between >4 and <7 indicate partial remission/relapse [12,13]. CGI-S scale scores > 2 indicate relapse [14,15]. With a large effect size for HAMD6 scores (Cohen’s d = 0.8), the estimated sample size required was 15 (paired *t*-test; 80% power, *p* = 0.05). 

We also considered the OW TMS and OF TMS groups separately. Some patients were in remission and others in partial remission/relapse—as these starting positions could possibly influence the clinical trajectory, we considered these as another two separate categories. Thus, four sub-groups were arranged for analysis: (a) OW in remission, (b) OW in partial remission/relapse, (c) OF in remission, and (d) OF in partial remission/relapse.

For each sub-group and the total patient sample, the mean ± SD were calculated for the following: (a) the number of weeks in therapy, (b) the admission HAMD6 and CGI-S scores, and (c) the HAMD6 and CGI-S scores across the treatment period. Differences within and between groups in HAMD6 and CGI-S scores were assessed with paired and unpaired *t*-tests, respectively. The effect size was determined by Cohen’s d. The correlation between changes in HAMD6 and CGI-S scores was assessed using Pearson’s correlation coefficient. A *p*-value of 0.05 (two-tailed) was considered statistically significant for all tests. The analyses were performed using SPSS (IBM Corp., IBM SPSS Statistics for Windows, version 27, 2020, Armonk, NY, USA).

When patients commenced treatment, they signed an agreement for their de-identified data to be used for clinical audit purposes. The St. Helens Private Hospital (Hobart, TAS, Australia) Medical Advisory Committee had approved the study and deemed it exempt from needing formal ethics approval. The TMS service had been provided within the hospital outpatient department before moving to a community clinic setting when the hospital campus closed.

## 3. Results

Thirteen patients were offered continuation TMS. One, Patient E, commenced in the OW TMS arm, but after 11 weeks elected to receive OF TMS. In many calculations, Patient E was necessarily counted twice, and we conceptualise 14 patients. The OW TMS group had 10 patients with a mean age of 51 years—8 females and 2 males, with a mean MDD history of 17 years (Table 1). The OF TMS group had 4 patients with a mean age of 48 years—2 females and 2 males, with a mean MDD history of 17 years (Table 2). 

No relapses occurred in patients initially in remission or partial remission. Three patients who felt well for some weeks, elected to withdraw from treatment [(a) Patient E received 11 OW treatments followed by 6 OF treatments (over 24 weeks), (b) Patient K received 5 OF treatments (over 8 weeks), and (c) Patient H received 9 weeks of OW treatments]. The quantitative findings from these patients were included with those who remained in treatment. 

When all patients were considered together, the initial HAMD6 score was 5.9 ± 1.9 and the mean score across the treatment (mean 21.5 ± 12.6 weeks) was 4.9 ± 1.2 (t = 2.73, *p* = 0.017; Cohen’s d = 0.63); the initial CGI-S score was 3.3 ± 0.7 and the mean score across treatment was 2.7 ± 0.5 (t = 4.29, *p* < 0.001; Cohen’s d = 0.99). The magnitude of changes in HAMD6 and CGI-S scores were significantly correlated within patients (r = 0.54, *p* < 0.05). The changes in HAMD6 and CGI-S scores were not significantly different between the OW and OF groups. 

As a group, the 10 OW TMS patients commenced with a mean HAMD6 score of 5.9 ± 2.1, and this was reduced to 4.9 ± 1.1 (t = 2.17, *p* = 0.058; Cohen’s d = 0.60) with treatment (mean 23.4 ± 13.4 weeks). The initial mean CGI-S score was 3.3 ± 0.7, with an improvement to 2.7 ± 0.6 (t = 5.07, *p* < 0.001; Cohen’s d = 0.92) with treatment. Those OW patients who commenced in remission had a mean admission HAMD6 score of 4.0 ± 0, and their mean score across treatment remained at 4.1 ± 0.5. Their admission CGI-S score was 2.8 ± 0.5 and across treatment mean score was 2.3 ± 0.3 (t = 3.18, *p* = 0.05; Cohen’s d = 1.21). Six patients commenced OW in partial remission/relapse with a mean admission HAMD6 score of 7.2 ± 1.7. Their mean score across treatment was 5.5 ± 1.1 (t = 2.82, *p* = 0.037; Cohen’s d = 1.19). Their admission CGI-S score was 3.7 ± 0.5 and their mean score across treatment was 3.0 ± 0.5 (t = 3.90, *p* = 0.012; Cohen’s d = 1.40). That is, their HAMD6 and CGI-S scores both significantly improved.

As a group, the 4 OF TMS patients commenced with a mean HAMD6 score of 5.8 ± 1.5, and this was reduced to 4.9 ± 1.4 (t = 1.64, *p* = 0.20; Cohen’s d = 0.62) with treatment (mean 16.8 ± 10.4 weeks). The initial mean CGI-S score was 3.3 ± 1.0, and subsequently 2.8 ± 0.5 (t = 1.23, *p* = 0.30; Cohen’s d = 0.63) with treatment. Only one OF patient commenced in remission. Their initial HAMD6 score was 4, and the mean score across treatment was 3.2. Their initial CGI-S score was 3 and the mean score across treatment score was 2.2. Three OF patients commenced in partial remission/relapse. Their mean admission HAMD6 score was 6.3 ± 1.2 and their mean score across the treatment was 5.5 ± 0.5. Their mean admission CGI-S was 3.3 ± 1.2 and their mean score across the treatment was 3.0 ± 0.5. That is, both HAMD6 and CGI-S scores improved, although these changes were not statistically significant with the small sample.

A sensitivity analysis was performed by excluding data from the three patients who felt well for some weeks and elected to withdraw from treatment. The key results were essentially unchanged. When the remaining eleven patients were considered together, the initial HAMD6 score was 5.8 ± 1.8 and the mean score across the treatment (mean 24.9 ± 12.1 weeks) was 5.0 ± 1.2 (t = 2.32, *p* = 0.04; Cohen’s d = 0.52); the initial CGI-S score was 3.4 ± 0.7 and the mean score across treatment was 2.7 ± 0.6 (t = 5.23, *p* < 0.001; Cohen’s d = 1.07). As a group, the remaining 9 OW TMS patients commenced with a mean HAMD6 score of 5.6 ± 1.9, and this was reduced to 4.9 ± 1.2 (t = 1.75, *p* = 0.12; Cohen’s d = 0.44) with treatment (mean 25.0 ± 13.2 weeks). The initial mean CGI-S score was 3.2 ± 0.7, with an improvement to 2.7 ± 0.5 (t = 4.46, *p* = 0.002; Cohen’s d = 0.82) with treatment.

## 4. Discussion

When patients were considered in sub-categories or as a heterogenous group, over an average of 21.5 weeks of maintenance treatment, there was no evidence of relapse, and no patient withdrew due to dissatisfaction or adverse effects. Additionally, there were improvements in HAMD6 and CGI-S scores that were statistically significant or of borderline statistical significance for the total sample and the OW group, with medium to large effect sizes. The small study suggests that OW TMS is effective as maintenance treatment. It also offers some support for OF TMS maintenance, at least in terms of preventing relapse.

As noted by Wilson et al. in their systematic review [3], the treatment-resistant patients for whom TMS is often provided have very limited treatment options, and maintenance TMS is a reasonable clinical option in these carefully selected patients based on current safety and effectiveness data.

Our finding that both OW and OF treatment can be effective in preventing relapse is consistent with controlled studies and a meta-analysis [7,8,9]. In contrast, a systematic review suggested that administering two or fewer TMS sessions per month may be ineffective in sustaining an antidepressant effect or in reducing the risk of relapse in responder patients [10]. Given the small sample in our OF treatment group and the statistically insignificant changes in the psychometric scale outcomes, we suggest that this form of maintenance needs further examination in larger groups and would presently give preference to OW TMS as a maintenance treatment. 

Limitations of the study should be acknowledged. It was not a randomised, controlled trial. It is possible that the observed improvements could be due to placebo effects or natural fluctuations in the course of MDD, although the treatment period was up to 46 weeks. The lack of blinding could have introduced bias in the assessment of outcomes. The number of participants was also small, especially for the OF group, limiting the statistical power. Given the limited numbers, we did not perform multivariate analyses to consider any potential confounding factors that may have influenced the response to TMS. However, this was a real-life study of patients who had suffered long-standing severe, relapsing MDD, and over half had previously received ECT. We did not formally assess patient functioning or quality of life. However, to maintain these individuals out of hospital and free of ECT over the mean treatment period of almost 6 months is evidence of a significant contribution to their quality of life (and that of their families). Of course, it should be borne in mind that the results in our sample may not be generalisable to all groups of patients with long-standing severe, relapsing MDD. Large randomised, controlled trials of maintenance TMS in this population are desperately needed, noting the practical difficulties involved, including that individuals who have already experienced a number of weeks of TMS as an acute treatment will readily appreciate the difference between how active and sham TMS feels [3].

## 5. Conclusions

This naturalistic, open-label observational study provides evidence suggesting that for patients with MDD who have a history of rapid relapse, OW TMS may be an effective means of maintaining remission. The method we have described involves more treatment sessions than some other continuation protocols. Currently, one group is studying 36 treatments over two years [18]. Our protocol would involve either 100 or 50 treatments over two years. Relapse of MDD may carry dire consequences—a possible route forward may be for OW (or perhaps OF in some individuals) maintenance TMS to be available and affordable for patients.

## Figures and Tables

**Table 1:** Patients who commenced OW TMS in remission and partial remission/relapse.

Patient	Sex	Age (yrs)	Age at Initial Onset (yrs)	Past ECT	Past Acute TMS	Past CM-TMS	Weeks in Treatment	Initial HAMD6	Mean HAMD6 in Treatment	Initial CGI-S	Mean CGI-S in Treatment
Commenced OW TMS in remission											
A	F	52	27	Y	Y	Y	46	4	4.2	3	2.2
B	F	38	33	N	Y	Y	37	4	3.3	2	1.9
C	F	76	50	N	Y	Y	40	4	4.3	3	2.6
D	M	52	30	Y	Y	Y	27	4	4.4	3	2.4
		Mean 54.5	Mean 35				Mean 37.5	Mean 4.0	Mean 4.1	Mean 2.8	Mean 2.3
Commenced OW TMS in partial remission or relapse											
E ^#^	M	42	34	Y	Y	Y	11	5	5.5	3	3
F	F	35	16	Y	Y	Y	22	6	4.1	3	2.2
G	F	64	40	N	Y	Y	16	6	4.7	4	2.8
H	F	44	30	Y	Y	Y	9	9	5.7	4	3.3
I	F	57	55	N	Y	N	12	8	7.2	4	3.6
J	F	54	25	N	Y	Y	14	9	6	4	3.2
		Mean 49.3	Mean 33.3				Mean 14.0	Mean 7.2	Mean 5.5	Mean 3.7	Mean 3.0

^#^ Transferred after 11 weeks of OW TMS, to OF TMS.

**Table 2:** Patients who commenced OF TMS in remission and partial remission/relapse.

Patient	Sex	Age (yrs)	Age at Initial Onset (yrs)	Past ECT	Past Acute TMS	Past CM-TMS	Weeks in Treatment	Initial HAMD6	Mean HAMD6 in Treatment	Initial CGI-S	Mean CGI-S in Treatment
Commenced OF TMS in remission											
K	F	47	30	Y	Y	Y	8	4	3.2	3	2.2
Commenced OF TMS in partial remission or relapse											
L	F	46	20	N	Y	Y	31	7	4.8	4	2.8
E ^#^	M	42	34	Y	Y	Y	10	5	5.2	2	2.6
M	M	56	39	Y	Y	Y	18	7	6.5	4	3.5
		Mean 48	Mean 31				Mean 19.7	Mean 6.3	Mean 5.5	Mean 3.3	Mean 3.0

^#^ Transferred to OF TMS after 11 weeks of OW TMS.

## Data Availability

The data presented in this study are available on request from the corresponding author due to the study being a clinical audit.

## References

[1] Sackeim H. (2016). Acute continuation and maintenance of major depressive episodes with transcranial magnetic stimulation. Brain Stimul..

[2] Psychotropic Expert Group (2021). Transcranial magnetic stimulation. Therapeutic Guidelines: Psychotropic.

[3] Wilson S., Croarkin P., Aaronson S., Carpenter L.L., Cochran M., Stultz D.J., Kozel F.A. (2022). Systematic review of preservation TMS that includes continuation, maintenance, relapse-prevention and rescue TMS. J. Affect. Disord..

[4] Fitzgerald P., Grace N., Hoy K., Bailey M., Daskalakis Z. (2013). An open label trial of clustered maintenance rTMS for patients with refractory depression. Brain Stimul..

[5] Pridmore S., Erger S., Rybak M., Kelly E., May T. (2018). Early relapse (ER) transcranial magnetic stimulation (TMS) in treatment resistant major depression. Brain Stimul..

[6] Philip N., Dunner D., Dowd S., Aaronson S., Brock D., Carpenter L.L., Demitrack M.A., Hovav S., Janicak P.G., George M.S. (2016). Can medication free, treatment-resistant, depressed patients who initially respond to TMS be maintained off medications? A prospective, 12-month multisided randomized pilot study. Brain Stimul..

[7] Benadhira R., Thomas F., Bouaziz N., Braha S., Andrianisaina P.S., Clémence Isaac C., Moulier V., Januel D. (2017). A randomized, sham-controlled study of maintenance rTMS for treatment-resistant depression (TRD). Psychiatry Res..

[8] Haesebaert F., Moirand R., Schott-Pethelaz A., Brunelin J., Poulet E. (2018). Usefulness of repetitive transcranial magnetic stimulation as a maintenance treatment in patients with major depression. World J. Biol. Psychiatry.

[9] Chen Y.-C.B., Chou P.-H., Tu Y.-K., Brunoni A., Kuan-Pin Su K.-P., Tseng P.-T., Liang C.-S., Lin P.-Y., Carvalho A.F., Hung K.-C. (2023). Trajectory of changes in depressive symptoms after acute repetitive transcranial magnetic stimulation: A meta-analysis of follow-up effects. Asian J. Psychiatry.

[10] d’Andrea G., Mancusi G., Santovito M.C., Marrangone C., Martino F., Santorelli M., Miuli A., Di Carlo F., Signorelli M.S., Clerici M. (2023). Investigating the Role of Maintenance TMS Protocols for Major Depression: Systematic Review and Future Perspectives for Personalized Interventions. J. Pers. Med..

[11] Pridmore S., Pridmore W. (2023). Medicare Benefits Schedule item numbers for transcranial magnetic stimulation (TMS): Questions arising. Australas. Psychiatry.

[12] Paykel E. (2008). Partial remission, residual symptoms, and relapse into depression. Dialogues Clin. Neurosci..

[13] Rush A.J., Kraemer H.C., Sackheim H.A., Fava M., Trivedi M.H., Frank E., Ninan P.T., Thase M.E., Gelenberg A.J., Kupfer D.J. (2006). Report by the ACVP Task force on response and remission in major depressive disorder. Neuropsychopharmacology.

[14] Bandelow B., Baldwin D., Dolberg O., Andersen H., Stein D. (2006). What is the threshold for systematic response and remission for major depressive disorder, panic disorder, social anxiety disorder, and generalized anxiety disorder?. J. Clin. Psychiatry.

[15] Busner J., Targum S. (2007). The clinical global impression scale. Psychiatry.

[16] Timmerby N., Andersen J.H., Søndergaard S., Østergaard S.D., Bech P. (2017). A systematic review of the clinimetric properties of the 6-item version of the Hamilton depression rating scale (HAM-D6). Psychother. Psychosom..

[17] Berk M., Ng F., Dodd S., Callaly T., Campbell S., Bernardo M., Trauer T. (2008). The validity of the CGI severity and improvement scales as measures of clinical effectiveness suitable for routine clinical use. J. Eval. Clin. Pract..

[18] Faruqui Z., Mania I., Gigilashvili M., Akubardia N. (2023). Long-term preservation transcranial magnetic stimulation for major depressive disorder. Prim. Care Companion CNS Disord..

